# Fish oil protects the blood–brain barrier integrity in a mouse model of Alzheimer’s disease

**DOI:** 10.1186/s13020-020-00314-0

**Published:** 2020-03-30

**Authors:** Youna Xie, Lingli Yan, Haitao Zeng, Weineng Chen, Jia-Hong Lu, Jian-Bo Wan, Huanxing Su, Xiaoli Yao

**Affiliations:** 1 Department of Neurology, The First Affiliated Hospital, Sun Yat-sen University, Guangdong Provincial Key Laboratory of Diagnosis and Treatment of Major Neurological Diseases, National Key Clinical, Department and Key Discipline of Neurology, No.58 Zhongshan Road 2, Guangzhou, 510080 China; 2 State Key Laboratory of Quality Research in Chinese Medicine, Institute of Chinese Medical Sciences, University of Macau, Macau, China; 3 Center for Reproductive Medicine, The Sixth Affiliated Hospital, Sun Yat-sen University, Guangzhou, 510080 People’s Republic of China

**Keywords:** Omega-3 polyunsaturated fatty acids, Aβ-degrading enzymes, Neuroinflammation, NF-κB, Amyloid beta

## Abstract

**Background:**

Alzheimer’s disease (AD) is ranked as the most prevalent neurodegenerative disease. However, the exact molecular mechanisms underlying pathophysiological alterations in AD remain unclear, especially at the prodromal stage. The decreased proteolytic degradation of Aβ, blood–brain barrier (BBB) disruption, and neuroinflammation are considered to play key roles in the course of AD.

**Methods:**

Male APPswe/PS1dE9 C57BL/6 J double-transgenic (APP/PS1) mice in the age range from 1 month to 6 months and age-matched wild type mice were used in this study, intending to investigate the expression profiles of Aβ-degrading enzymes for Aβ degradation activities and zonula occludens-1 (zo-1) for BBB integrity at the prodromal stage.

**Results:**

Our results showed that there were no significant genotype-related alterations in mRNA expression levels of 4 well-characterized Aβ-degrading enzymes in APP/PS1 mice within the ages of 6 months. Interestingly, a significant decrease in zo-1 expression was observed in APP/PS1 mice starting from the age of 5 months, suggesting that BBB disrupt occurs at an early stage. Moreover, treatment of fish oil (FO) for 4 weeks remarkably increased zo-1 expression and significantly inhibited the glial activation and NF-κB activation in APP/PS1 mice.

**Conclusion:**

The results of our study suggest that FO supplement could be a potential therapeutic early intervention for AD through protecting the BBB integrity and suppressing glial and NF-κB activation.

## Background

Alzheimer’s disease (AD) is the most common neurodegenerative disorder among older population worldwide. Pathological hallmarks of AD include extracellular amyloid plaques and intracellular neurofibrillary tangles in brain regions [1, 2]. A large body of studies suggest that amyloid plaques result from an imbalance between production and clearance of amyloid beta (Aβ) [3, 4]. Aβ can be cleared by a number of pathways in the brain such as enzymatic degradation and recirculation into the blood stream via the blood–brain barrier (BBB) [5]. In the past decades, a series of degrading enzymes have been identified to be able to cleave Aβ either in vitro or in vivo, including cathepsin B (CatB), neprilysin (NEP), insulin-degrading enzyme (IDE), and myelin basic protein (MBP). CatB is specifically implicated as a proteolytic enzyme in degradation of Aβ in vivo [6]. NEP is reported to degrade both monomeric and oligomeric Aβ [7], IDE is concentrated on soluble monomeric clearance [8], and MBP can degrade both monomeric and fibrillary forms of Aβ [9]. Aβ deposit is found not just in AD patients but even in cognitively normal elderly [10], suggesting that Aβ enzymatic degradation might be impaired at the early disease stage. Therefore, it is of interest to investigate the expression profiles of Aβ-degrading enzymes at the prodromal stage of AD.

The BBB acts to limit the molecules exchange between brain and periphery regions mainly relying on tight junction (TJ) structure in BBB endothelial cells, of which destruction could alter brain homeostasis, and lead to brain edema, neuroinflammation, neuron injury, and so on [11]. Zonula occludens-1 (zo-1), as a TJ periphery membrane protein, belongs to the membrane-associated guanylate kinase (MAGUK) family. Zo-1 is a tight junction (TJ) protein, which is essential for maintaining and stabilizing the TJ structure. Decrease of zo-1 could result in BBB integrity injury, which could disturb Aβ clearance via Aβ transporters on BBB and alter brain homeostasis that leads to brain-wide neuroinflammation and neuronal injury [12–15]. Previous studies reported that the damage of BBB integrity participated in AD pathology [16, 17]. The studies by Kook et al. and Wan et al. suggested that oligomeric Aβ_1–42_ could trigger the decrease of tight junctions and result in barrier integrity injury [18, 19]. Indeed, BBB integrity alterations were detected in the quite early stage even before amyloid plaques deposit and cognitive deficits in AD transgenic mice [20, 21]. Several attempts focusing on protecting BBB function have shown positive effects on AD pathology, suggesting that the BBB could be a potential therapeutic target for AD.

Many studies have demonstrated that omega-3 polyunsaturated fatty acids (ω3-PUFAs) confer benefits in a variety of neurological disorders due to their anti-inflammatory, anti-oxidant, anti-apoptotic and neuroprotective effects [22–28]. Evidence from a preclinical study suggested that dietary intervention with ω3-PUFAs could reduce AD risk [29]. Moreover, Docosahexaenoic acid (DHA), a major member of long-chain ω3-PUFAs, was found to reduce Aβ deposit in an aged AD mouse model [30]. Very recently, we have reported that ω3-PUFAs could promote glymphatic function to enhance Aβ clearance from the brain [31]. However, clinical research suggested that DHA supplement improved cognition in patients with mild cognition impairment but not AD patients with severe cognition impairment [32], which indicates the importance of an early intervention.

In the present study, we firstly investigated the expression profiles of Aβ-degrading enzymes for Aβ degradation activities and zo-1 for BBB permeability in APP/PS1 transgenic mice at the prodromal stage (prior to the age of 6 months). Our results showed that there were no significant genotype-related alterations in mRNA expression of 4 well-characterized Aβ-degrading enzymes in APP/PS1 mice within the ages of 6 months while there was a significant decrease in zo-1 expression starting from the age of 5 months. We further demonstrated that treatment of fish oil (FO) for 4 weeks remarkably increased zo-1 expression and significantly inhibited the glial activation and NF-κB activation in APP/PS1 mice.

## Methods

### Animal

APPswe/PS1dE9 C57BL/6 J double-transgenic (APP/PS1) mice at different ages ranging from 1-month to 6-month old and age-match wild type (WT) mice were used in the present study. Male heterozygous APP/PS1 mice were obtained from Jackson Laboratory (Bar Harbor, ME, USA) and used to mate with female C57BL/6 mice to generate heterozygous mice and WT littermates for this research. The genotypes of APP/PS1 animals were identified by standard polymerase chain reaction (PCR) analysis of genomic DNA isolated from mouse tails. All mice were housed in groups and accessed to food and water ad libitum in a 12:12 h light–dark (light on at 8: 00 am) cycle, provided with controlled temperature and humidity.

### FO administration

APP/PS1 mice at 4-month and 5-month old received daily intragastrical administration of FO. Each animal received 50 μL FO (containing 13 μM Eicosapentaenoic Acid (EPA) and 99 μM DHA; the purity of EPA + DHA = 80.27%; Wuhan Shengtianyu Biotech Ltd., China) per day lasting for 4 weeks. EPA and DHA were suspended in corn oil which is free from contaminants. The main constituents of corn oil are oleic acid and linoleic acid, which are essential dietary elements for humans. Corn oil is widely used as a safe vehicle in the study of omega-3 polyunsaturated fatty acids. APP/PS1 mice in the control group received daily intragastrical administration of equivoluminal isocaloric corn oil (CO). Age-matched WT mice which received a normal diet served as a normal control.

### Fatty acid analysis

To evaluate the effects of the dietary regime on the PUFA composition in the brain, the hippocampal and cortical tissue samples of APP/PS1 mice from fish oil-and corn oil-treated groups (n = 3 per group) were processed for fatty acid analysis by gas chromatography-mass spectrometry (GC–MS) as described previously [33]. Quantifications were performed by an investigator who was blind to the animal grouping and carried out by normalizing individual peak areas as the percentage of total fatty acids.

### Tissue preparation

APP/PS1 and WT mice at the age of 1, 2, 3, 4, 5, and 6 months were sacrificed under overdose anesthetic (n = 3 at each time point). After transcardially perfused with ice-cold normal saline, the animal brains were quickly moved out to be placed on ice and then divided into two halves along the middle sagittal sulcus. The cortex and hippocampus of the right half were used to detect the expression of Aβ degrading enzymes by using real-time PCR (RT-PCR), and the cortex and hippocampus of the left half were used to measure the expression of zo-1 by western blot. APP/PS1 mice aged 5 months which received either FO or CO administration for 4 weeks were sacrificed and their brains were isolated for immunohistochemistry (5 mice each group) and western blot analysis (5 mice each group).

### Real-time PCR analysis

For isolating RNA from the cortex and hippocampus, TRIzol reagents (Catlog No: RN 190, Thermo Fisher Scientific, USA) were used under the manufacturer’s instructions, followed by RNA quality evaluation with a microplate reader (Thermo Fisher Scientific, USA). Two micrograms of RNA were reversely transcribed using a reverse transcription system kit under the manufacturer’s protocol. RT-PCR was performed in a 20 μL reaction system containing 10 μL TB Green Mix (RR820Q, TB GreenTM premix Ex TaqTM, Kapa Biosystems, USA), 2 μL of cDNA (diluted 20 times with de-enzyme water), 6.6 μL ddH2O, and 0.4 μL 500 nM of each specific primer. The cycling parameters were as follows: 95 °C, 300 s; 95 °C, 10 s; 60 °C, 40 s; 95 °C,10 s; 60 °C, 30 s; and 95 °C, 30 s. The primers used for PCR were shown as follows: CatB, forward: 5′-AAATCAGGAGTATACAAGCATGA-3′, reverse: 5′-GCCCAGGGATGCGGATGG-3′; NEP, forward: 5′- TCCTGACTATCATAGCGGTGAC-3′, reverse: 5′- GACGTTGCGTTTCAACCAGC-3′; IDE, forward: 5′-ACTAACCTGGTGGTGAAG-3′, reverse: 5′- GGTCTGGTATGGGAAATG -3′; MBP, forward:5′-CGGACCCAAGATGAAAACCC-3′, reverse: 5′-AAAGGAAGCCTGGACCACACAG-3′; GADPH, forward: 5′ AACGACCCCTTCATTGAC -3′; reverse:5′- TCCACGACATACTCAGCAC -3′.

### Western blot analysis

Proteins of cortex and hippocampus were extracted with lysis buffer containing a protease inhibitor, and a BCA protein assay kit (Catlog No: 23225, Thermo scientific, USA) was used for protein concentration analysis. After separated by electrophoresis on SDS-PAGE gel, sample proteins were transferred onto polyvinylidene fluoride membranes (Catlog No: 1621077, BIO-RAD, USA). The membranes were then incubated with primary and secondary antibodies, followed by incubation with enhanced chemiluminescence solution (Catlog No: RPN2235, GE Healthcare, Sweden) and autoradiography. Primary antibodies included anti-zo-1 (Catlog No: AB2272, EMD Millipore, USA), anti-NFκB-p65 (Catlog No: 8242, cell signaling technology, USA), anti-phosphorylated p65 (p-p65) (Ser536, Catlog No: 3303, cell signaling technology, USA), anti-tubulin (Catlog No: RM2007, Ray Antibody, China), and anti-actin (Catlog No: SC69879, Santa Cruz Biotechnology, USA). All results from chemiluminescence exposure were analyzed with software Image Pro plus 6.0.

### Immunofluorescence

Brain tissues post-fixed in 4% PFA were transferred into 20–30% sucrose buffer for 2 days at 4 °C, and were cut into 15 μm coronal sections. Selected coronal sections were incubated with primary antibodies anti-GFAP (Catlog No: 60190, sigma, USA) and anti-Iba-1 (Catlog No: 019-19741, Wako, Japan) overnight after blocked with 10% normal donkey serum (Beyotime technology) at room temperature for 1 h. Species-specific secondary antibodies were then added to the brain sections for 1 h at 37 °C in dark after washed with 0.01 M PBS 3 times. Following washing with 0.01 mol/l PBS, the sections were mounted using Fluoroshield Mounting Medium with DAPI (Catlog No: F6057, Sigma-Aldrich; Merck KGaA, Darmstadt, Germany). The expression of Iba-1 and GFAP was examined using the Olympus fluorescence microscope (20× magnification). Quantification of GFAP labelled astrocytes and Iba-1 labelled microglia was performed using the image analysis programme ImageJ (ImageJ 1.39u, National Institute of Health). For evaluating the average fluorescent density, 3 measuring frames of 1360 × 1024 pixels per section and a total of 5 randomly selected sections per animal were analyzed in a blinded manner by two investigators. The level of immunoreactivity was manifested as the ROI per view that contained immunoreactivity. The image acquirements and data quantifications were performed by an investigator who was blind to the experiment.

### Statistical analysis

All data were presented as mean ± SEM, and the graphs were made using GraphPad Prism 6.0. Two-tail student’s t test and one-way ANOVA followed by Turkey post hoc test were used for two-group and multiple-group comparisons respectively. The significant difference level was set 0.05 in all comparisons with SPSS 24.0.

## Results

### Aβ-degrading enzymes showed no declines before AD onset in APP/PS1 mice

**Fig. 1:**
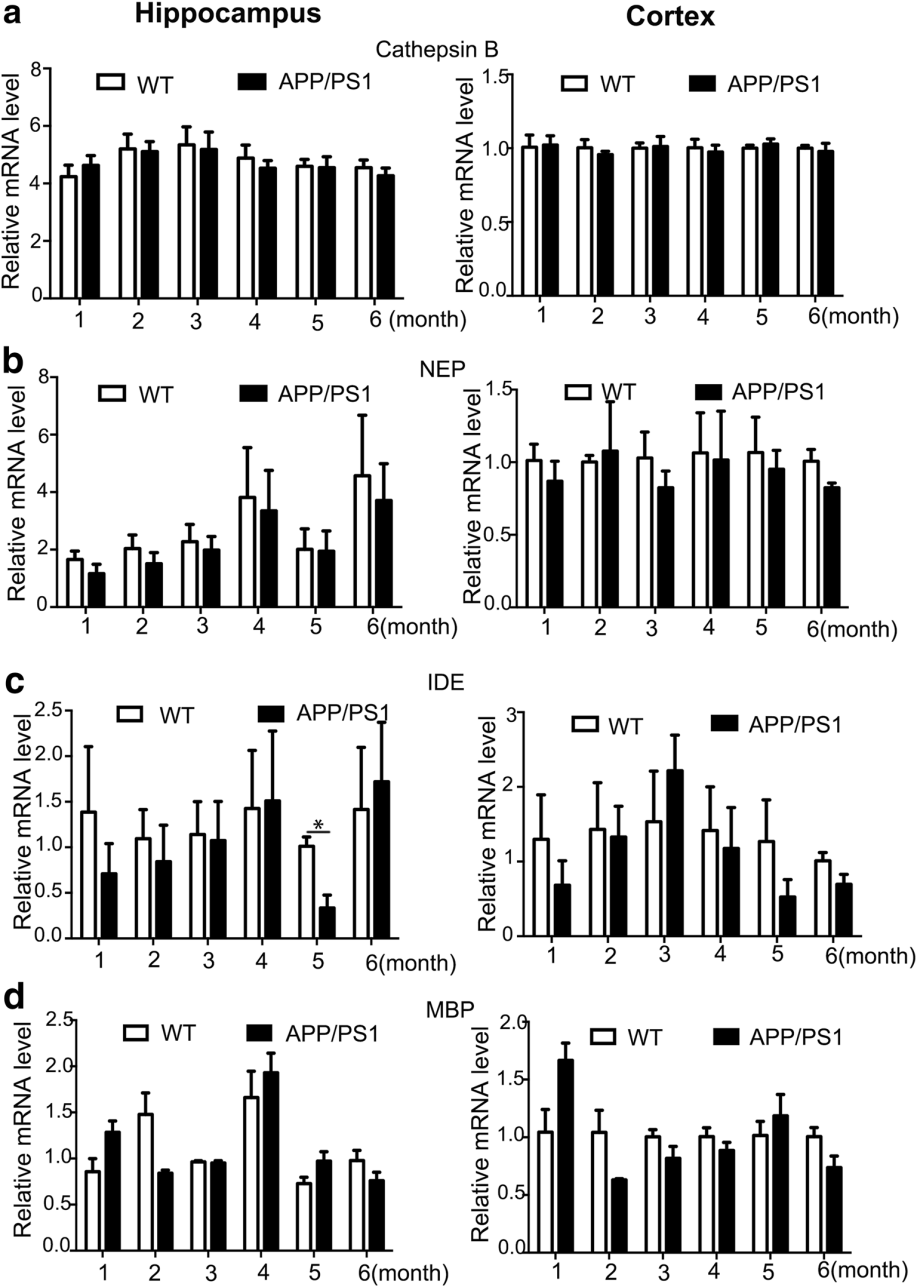
No alterations in mRNA expression of Aβ-degrading enzymes before AD onset in APP/PS1 mice. **a**–**d** Real-time PCR analysis revealed that no differences in mRNA expression level of were observed in the hippocampus and cortex in APP/PS1 mice aged from 1 to 6 months old compared to aged matched WT mice except for a decreased expression of IDE in the hippocampus of APP/PS1 mice compared to WT mice at the age of 5 months. A: CatB; B: NEP; C: IDE; and D: MBP. (*P < 0.05; n = 3 per group)

RT-PCR analysis was performed to assess whether there were alterations in mRNA expression of 4 well-identified Aβ-degrading enzymes including CatB, NEP, IDE, and MBP in APP/PS1 mice at the prodromal stage. No continuous alterations in the expression of CatB, NEP, IDE, and MBP were detected in both the hippocampus and the cortex of APP/PS1 mice within the early 6 months (Fig. 1a–d). Furthermore, no alterations on the mRNA expression level of CatB, NEP, IDE, and MBP were found in APP/PS1 mice compared to age-matched WT mice at the age of 1, 2, 3, 4, 5, or 6 months, in both the hippocampus and the cortex (Fig. 1a–d), except for a transient decrease in the expression of IDE in the hippocampus of APP/PS1 mice compared to WT mice at the age of 5 months (Fig. 1c).

### FO treatment reversed the declined expression of zo-1 before AD onset in APP/PS1 mice

**Fig. 2:**
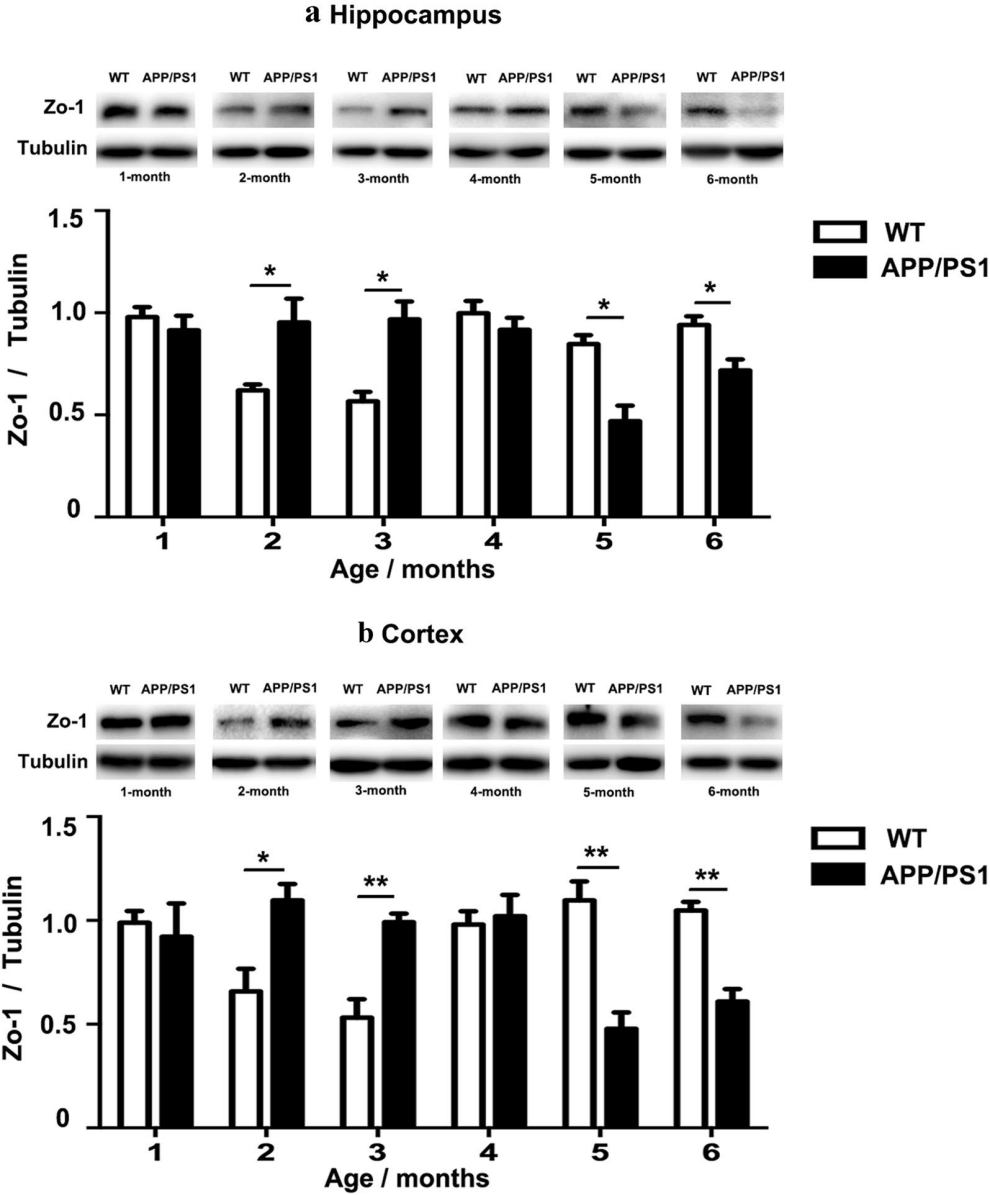
Changes of zo-1 level enzymes before AD onset in APP/PS1 mice. **a** Western blot revealed that zo-1 level was enhanced in the hippocampus of APP/PS1 mice compared to WT at 2- and 3-month old, whereas zo-1 expression level decreased in 5- and 6-month old APP/PS1 mice compared to age-match WT mice *P < 0.05; n = 3 per group). **b** Western blot revealed that zo-1 level was enhanced in the cortex of APP/PS1 mice compared to WT at 2- and 3-month old, whereas zo-1 expression level decreased in 5- and 6-month old APP/PS1 mice compared to age-match WT mice (*P < 0.05, **P < 0.01; n = 3 per group)

Changes of zo-1 level in both the hippocampus and cortex in APP/PS1 mice before disease onset were evaluated with Western blot analysis. There was no difference in the expression level of zo-1 between APP/PS1 and WT mice at the age of 1 month (Fig. 2a, b). An unexpected increase in the expression level of zo-1 in 2- and 3-month APP/PS1 mice was found as compared to their age-match WT mice (Fig. 2a, b). A significant decrease in zo-1 expression level was found in both the hippocampus and cortex of APP/PS1 mice at the age of 5 and 6 months compared to their age-matched WT mice (Fig. 2a, b).

**Fig. 3:**
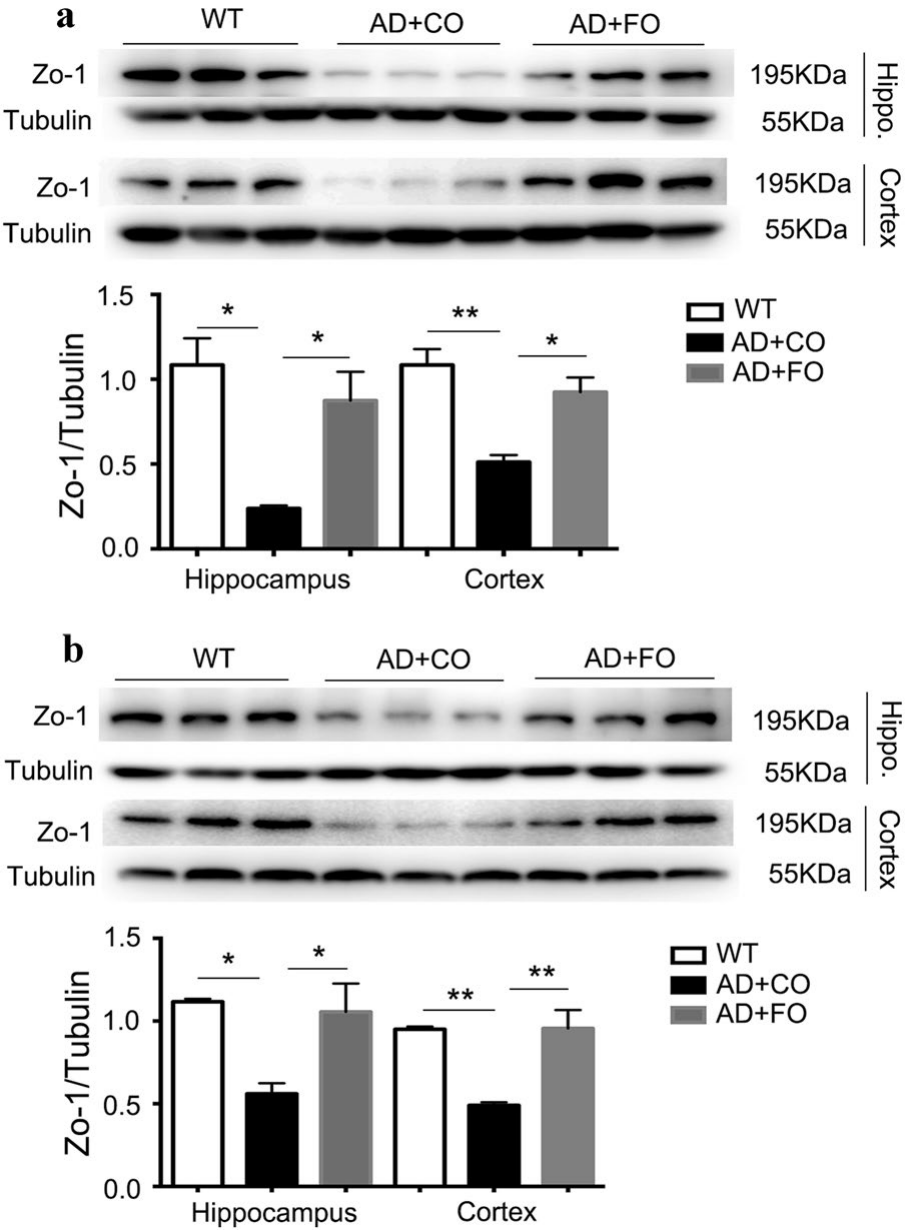
FO treatment reversed the declined expression of zo-1 in APP/PS1 mice. **a** Western blot analysis showed that FO treatment for 4 weeks significantly increased the expression levels of zo-1 in both the hippocampus and cortex of 4-month old APP/PS1 mice compared to CO-treated control animals (**P < 0.01; n = 3 per group). **b** Western blot analysis showed that FO treatment for 4 weeks significantly increased the expression levels of zo-1 in both the hippocampus and cortex of 5-month old APP/PS1 mice compared to CO-treated control animals (**P < 0.01; n = 3 per group)

There is evidence suggesting that ω3-PUFAs play an active role in the maintenance of biological membranes integrity and homeostasis [34]. To investigate whether treatment with FO (rich in ω3-PUFAs) could protect BBB integrity and permeability, we treated 4- and 5-month old APP/PS1 mice for 4 weeks. GC–MS showed that the expression level of ω-3 docosahexaenoic acid (DHA) and docosapentaenoic acid (DPA) in the hippocampus and cortex of APP/PS1 mice treated with fish oil for 4 weeks was significantly higher than that in the control group treated with corn oil (Tables 1, 2; n = 3 per group). Accordingly, the ratio of ω-6/ω-3 PUFAs was significantly lower in the fish oil-treated group compared with the corn oil-treated group (Tables 1, 2; n = 3 per group). Western blot analysis showed that FO treatment significantly increased the expression levels of zo-1 in both the hippocampus and cortex of APP/PS1 mice at the age of 5 and 6 months compared with CO-treated APP/PS1 mice (Fig. 3a, b).

**Table 1:** Profiles of polyunsaturated fatty acids in the hippocampus from fish oil-treated mice and the control mice with corn oil treatment (n = 3 per group)

Fatty acid	Hippocampus	
	Corn oil-mice	Fish oil-mice
ω-6 PUFA		
LA (C18:2 ω-6)	0.91 ± 0.02	0.91 ± 0.05
EDA (C20:2 ω-6)	0.27 ± 0.05	0.24 ± 0.03
DGLA (C20:3 ω-6)	0.51 ± 0.03	0.47 ± 0.05
AA (C20:4 ω-6)	13.91 ± 0.42	11.11 ± 1.02*
DTA (C22:4 ω-6)	4.26 ± 0.17	3.99 ± 0.21
n-6 DPA (C22:5 ω-6)	4.91 ± 0.43	1.26 ± 0.33***
Total	28.05 ± 1.12	18.41 ± 1.16*
ω-3 PUFA		
ω-3 DPA (C22:5 ω-3)	0.15 ± 0.01	0.39 ± 0.03**
DHA (C22:6 ω-3)	16.12 ± 1.89	27.18 ± 2.25**
Total	16.57 ± 0.44	26.52 ± 1.21**
Total PUFA	35.75 ± 1.59	43.01 ± 1.73*
ω-6/ω-3	1.56 ± 0.06	0.68 ± 0.06**

Data expressed as mol% of total fatty acids ± SEM (*P < 0.05; **P < 0.01; ***P < 0.001). *AA* arachidonic acid, *DGLA* dihomo-γ-linolenic acid, *DHA* docosahexaenoic acid, *DPA* docosapentaenoic acid, *DTA* docosatetraenoic acid, *EDA* eicosadienoic acid, *LA* linoleic acid, *MUFA* monounsaturated fatty acids (the value is given as follows: C16:1 + C18:1 + C20:1 + C22:1), *SFA* saturated fatty acids(the value is given as follows: C14:0 + C15:0 + C16:0 + C17:0 + C18:0 + C20:0 + C22:0 + C24:0); *PUFA* polyunsaturated fatty acids

**Table 2:** Profiles of polyunsaturated fatty acids in the cortex from fish oil-treated mice and the control mice with corn oil treatment (n = 3 per group)

Fatty acid	Cortex	
	Corn oil-mice	Fish oil-mice
ω-6 PUFA		
LA (C18:2 ω-6)	0.87 ± 0.07	0.89 ± 0.05
EDA (C20:2 ω-6)	0.29 ± 0.06	0.26 ± 0.03
DGLA (C20:3 ω-6)	0.57 ± 0.02	0.53 ± 0.04
AA (C20:4 ω-6)	16.21 ± 0.18	13.02 ± 0.65*
DTA (C22:4 ω-6)	4.23 ± 0.22	3.99 ± 0.15
n-6 DPA (C22:5 ω-6)	4.89 ± 0.45	1.69 ± 0.25***
Total	27.87 ± 1.35	20.76 ± 1.31*
ω-3 PUFA		
ω-3 DPA (C22:5 ω-3)	0.14 ± 0.01	0.39 ± 0.01**
DHA (C22:6 ω-3)	15.82 ± 1.02	25.05 ± 1.32**
Total	17.12 ± 0.58	27.21 ± 1.28**
Total PUFA	34.26 ± 1.45	44.26 ± 1.65*
ω-6/ω-3	1.89 ± 0.07	0.61 ± 0.09**

Data expressed as mol% of total fatty acids ± SEM (*P < 0.05; **P < 0.01; ***P < 0.001). *AA* arachidonic acid, *DGLA* dihomo-γ-linolenic acid, *DHA* docosahexaenoic acid, *DPA* docosapentaenoic acid, *DTA* docosatetraenoic acid, *EDA* eicosadienoic acid, *LA* linoleic acid, *MUFA* monounsaturated fatty acids (the value is given as follows: C16:1 + C18:1 + C20:1 + C22:1), *SFA* saturated fatty acids(the value is given as follows: C14:0 + C15:0 + C16:0 + C17:0 + C18:0 + C20:0 + C22:0 + C24:0), *PUFA* polyunsaturated fatty acids

### FO treatment inhibited glial activation and NF-κB activation in APP/PS1 mice

**Fig. 4:**
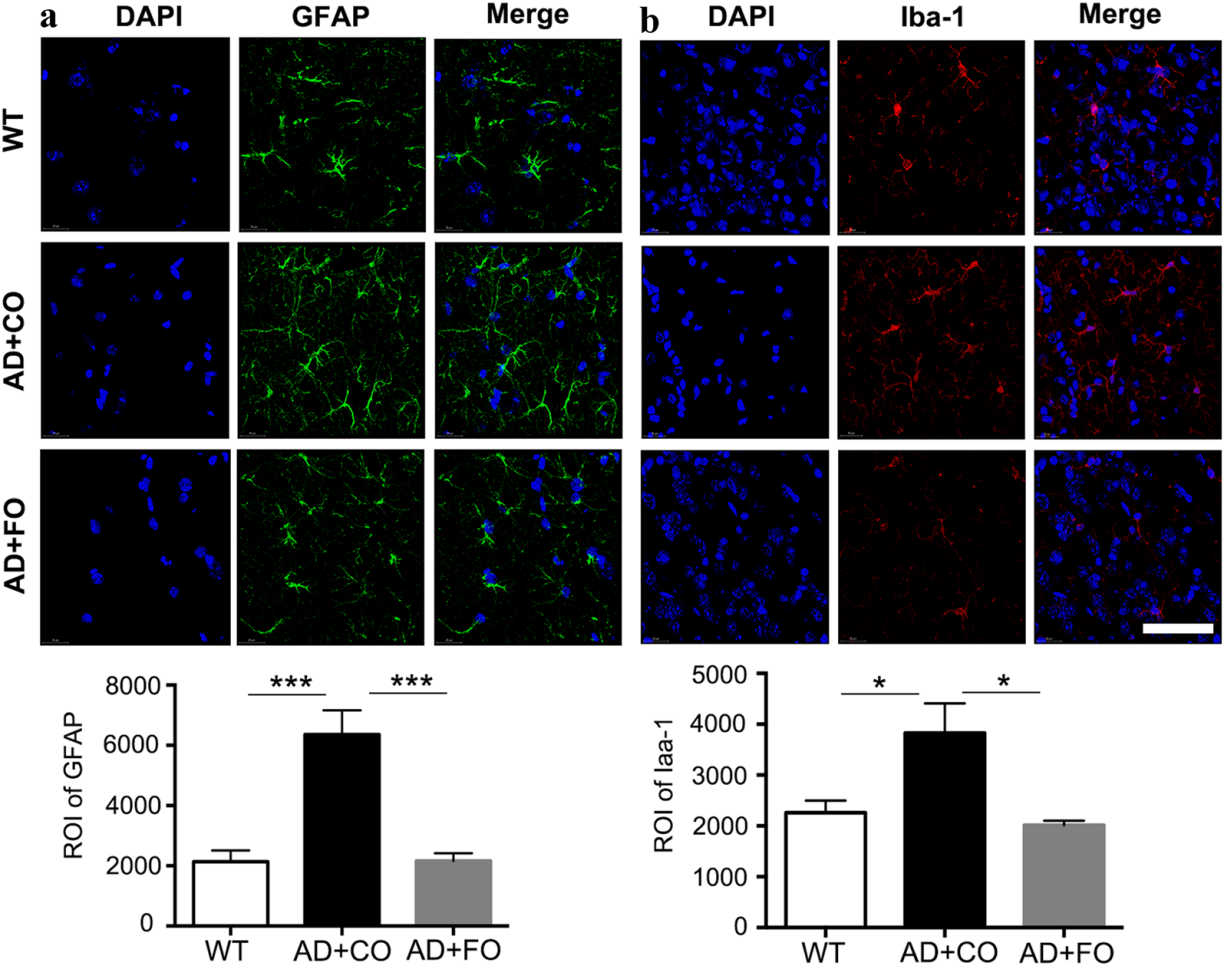
FO treatment inhiited the glial activation in APP/PS1 mice. **a** Representative images of GFAP expression in the cortex of the 5-month old APP/PS1 mice treated with FO or CO for 4 weeks and the age-matched WT mice (normal control). Higher relative optical intensity (ROI) of GFAP staining was observed in the cortex of APP/PS1 mice treated with CO compared to the WT mice, whereas FO treatment significantly suppressed astroglial activation in APP/PS1 mice compared to CO treatment (***P < 0.001; n = 5 per group). **b** Representative images of Iba-1 expression in the cortex of the 5-month old APP/PS1 mice treated with FO or CO for 4 weeks and the age-matched WT mice (normal control). Higher relative optical intensity (ROI) of Iba-1 staining was observed in the cortex of APP/PS1 mice treated with CO compared to the WT mice, whereas FO treatment significantly suppressed microglial activation in APP/PS1 mice compared to CO treatment (*P < 0.05; n = 5 per group). Scale bar: 75 µm

It is widely considered that neuroinflammation is implicated in AD pathology. For example, microglial activation triggered by Aβ could be observed in early AD [35, 36]. As results, pro-inflammation factors released from activated microglia then triggered complicated cascade reactions, contributing to the disease development. We treated 5-month old APP/PS1 mice with either FO or CO for 4 weeks. FO treatment significantly inhibited glial activation of the cortex region, which was demonstrated by lower relative optical intensity (ROI) of GFAP and Iba-1 staining found in the cortex of FO-treated APP/PS1 mice compared to the CO-treated APP/PS1 mice (Fig. 4a, b).

**Fig. 5:**
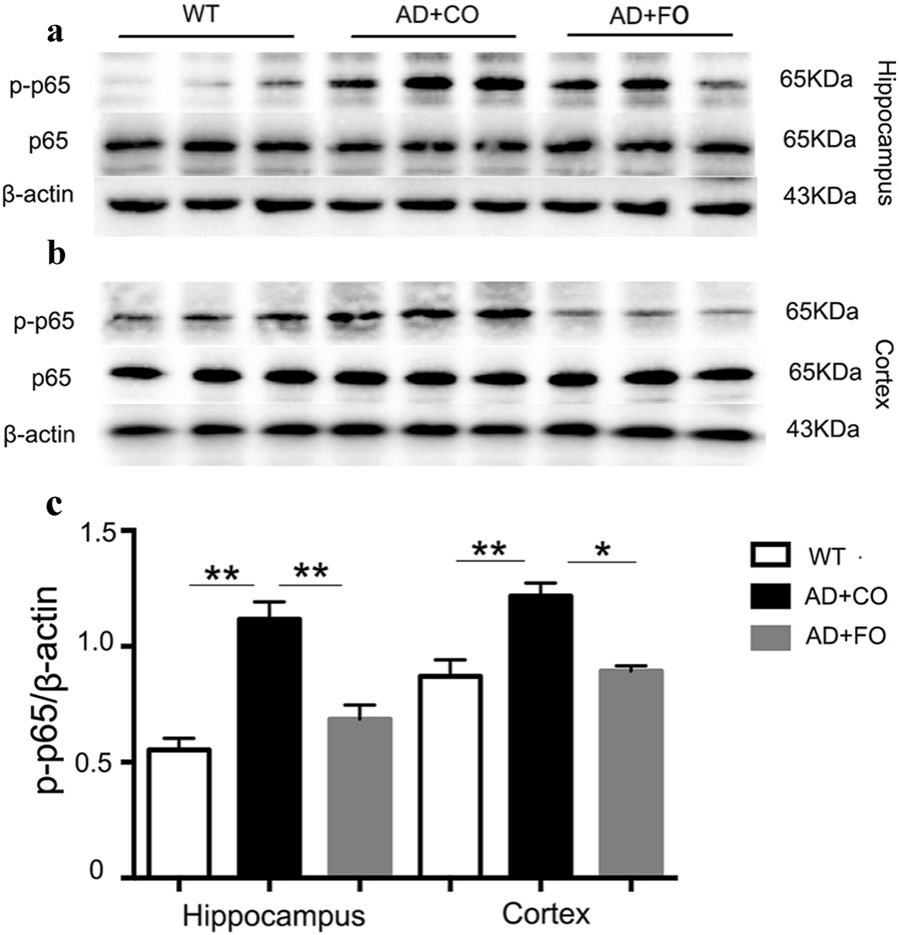
FO treatment inhibited NF-κB activation in both the hippocampus and cortex region of the APP/PS1 mice. **a** Representative images of Western blot analysis on the expression of phosphorylated p65 (p-p65, Ser536) and total p65 in the hippocampus region of the 5-month old APP/PS1 mice treated with FO or CO for 4 weeks and the age-matched WT mice (normal control). **b** Representative images of Western blot analysis on the expression of phosphorylated p65 (p-p65, Ser536) and total p65 in the cortex region of the 5-month old APP/PS1 mice treated with FO or CO for 4 weeks and the age-matched WT mice (normal control). **c** Quantification analysis revealed that FO treatment significantly suppressed the NF-κB activation found in APP/PS1 mice (*P < 0.05, **P < 0.01; n = 5 per group)

NF-κB activation is a key regulator for stimulating pro-inflammatory gene transcription [37]. Normally p65 combines with p50 as heterodimers in the cytoplasm, and its activity is inhibited by IκB. When IκB is activated and phosphorylated, it will stop suppressing the p65:p50 dimers. The released p65 will be phosphorylated and translocated into the nucleus where it binds with the target DNA and triggers a series of inflammatory genes transcription such as IL-1β, TNF-α, and NLRP3 [38, 39]. As the results showed, a significant elevation in the expression of phosphorylated p65 was observed in both the hippocampus and cortex of the APP/PS1 mice treated with CO compared to WT mice, suggesting that the NF-κB signaling pathway was activated in APP/PS1 mice (Fig. 5a–c). FO treatment significantly suppressed NF-κB activation in APP/PS1 mice as compared to the CO-treated group (Fig. 5a–c).

## Discussion

During the last decade, failure of clinical trials for AD was announced in succession [40–42]. Most of these clinical trials have focused on strategies for treating AD patients with developed symptoms. The failure of these clinical trials suggests that it might be too late to treat AD and an effective treatment for AD might need an early intervention. Exact molecular mechanisms underlying pathophysiological alterations in AD still remain unclear. The amyloid cascade hypothesis is one of the most well-known molecular mechanisms underlying pathophysiological alterations in AD [43]. This hypothesis proposes that Aβ deposits trigger neuronal dysfunction and death in AD.

Accumulating evidence suggests that abnormal Aβ deposits result from an imbalance between production and clearance of Aβ [3, 4]. While pharmacologic inhibition targeted at Aβ production could be effective in reducing Aβ accumulation, Aβ clearance is another effective way to reduce Aβ levels in the brains of AD. A series of Aβ-degrading enzymes have been identified which play key roles in determining cerebral Aβ levels under either physiological or pathophysiological conditions. NEP is one of the principal Aβ-degrading proteases and is the first one identified to be able to efficiently degrade Aβ in animal models [44]. NEP and IDE levels were aberrantly decreased at the dementia stage of AD patients, but not in the preclinical stage of AD patients, and the same results were also found in the APP/PS1 mice [45–47], suggesting that Aβ-degrading activities are gradually reduced after disease onset in AD. Interestingly, plaques burden decrease was obviously observed in 14-month APP transgenic mice which genetically overexpressed NEP or IDE [48]. In our study, the expression levels of 4 well-characterized Aβ-degrading enzymes did not decrease at the prodromal stage of AD mice. It was reported that APPSwe/PSEN1(dE9) mice showed contextual memory deficit at the early age of 6 months, while spatial memory impairment occurred at 8–10 months old [49–51]. Our results indicated that therapeutic approaches based on Aβ degradation could be designed for reducing cerebral Aβ levels after disease onset.

Several studies have reported that BBB disruption is identified as one of AD pathogeneses [52, 53]. Zo-1, one of the most important tight junctions of BBB, plays an important role in BBB integrity maintenance [54]. Our study demonstrated that the decrease of zo-1 expression in APP/PS1 mice started from 5-month-old, suggesting that the integrity of BBB alters at a quite early stage. Remarkably, FO treatment could counteract the decreased expression of zo-1 when applied to APP/PS1 mice, which provides evidence that ω3-PUFAs could be used as an early intervention agent for protecting BBB integrity in AD. An unexpected elevation of zo-1 expression in 2- and 3- month APP/PS1 mice was observed, but the exact mechanism for this temporary increase remains unknown. We propose that the abnormal production of Aβ in APP/PS1 mice at such an early stage may stimulate the expression level of zo-1 to compensate the dynamic of BBB as previously reported [55].

Neuroinflammation has been well defined in AD and considered as one of crucial AD pathogeneses [56–58]. Aβ deposition and Tau fragment could trigger a cascade inflammation [59, 60]. Our study showed that NF-κB was activated APP/PS1 mice at the age of 5 and 6 months. The activated NF-κB is essential for both acute and chronic inflammatory responses. It is widely accepted that ω3-PUFAs and their metabolites such as Resolvin D1 and Neuroprotectin D1 could function as potent anti-inflammatory molecules that suppresses inflammation and helps in the resolution of inflammatory events. Our findings demonstrated that FO supplement could be an early intervention for AD through significantly suppressing glial activation and NF-κB activation. In the pathogenesis of AD, Aβ accumulation induces the glial cell activation and impairs the BBB integrity. Interestingly, the impairment of BBB integrity activates glial cells to secret inflammatory factors, which in turn induces Aβ deposition in the brain of AD [61, 62]. Fish oil shows effects on protecting the BBB integrity and suppressing glial and NF-κB activation, suggesting that fish oil supplement is a promising therapeutic early intervention for AD.

## Conclusions

In summary, our study investigated Aβ enzymatic degradation activities and BBB integrity in APP/PS1 mice at the prodromal stage. No significant genotype-related alterations in mRNA expression of Aβ-degrading enzymes were found in APP/PS1 transgenic mice within the ages of 6 months, whereas the protein expression level of zo-1 decreased in 5- and 6-month APP/PS1 transgenic mice compared to their age-matched WT mice. Our study provides evidence that FO supplement could be a potential therapeutic early intervention for AD through protecting the BBB integrity and significantly suppressing glial activation and NF-κB activation.

## Data Availability

Please contact corresponding authors for data requests.

## Abbreviations

AD: Alzheimer’s disease
Aβ: Amyloid beta
BBB: Blood–brain barrier
CatB: Cathepsin B
CO: Corn oil
DHA: Docosahexaenoic acid
EPA: Eicosapentaenoic acid
FO: Fish oil
IDE: Insulin-degrading enzyme
MBP: Myelin basic protein
NEP: Neprilysin
ω3-PUFAs: Omega-3 polyunsaturated fatty acids
PCR: Polymerase chain reaction
RT-PCR: Real-time PCR
TJ: Tight junction
WT: Wild type
zo-1: Zonula occludens-1
